# Use of Hydrolyzed Chinese Gallnut Tannic Acid in Weaned Piglets as an Alternative to Zinc Oxide: Overview on the Gut Microbiota

**DOI:** 10.3390/ani11072000

**Published:** 2021-07-05

**Authors:** Junying Sun, Kaijun Wang, Baichang Xu, Xiaomin Peng, Beibei Chai, Siwei Nong, Zheng Li, Shuibao Shen, Hongbin Si

**Affiliations:** 1 College of Animal Science and Technology, Guangxi University, Nanning 530004, China; 1818393033@alu.gxu.edu.cn (J.S.); kj-wang@foxmail.com (K.W.); 1918393057@st.gxu.edu.cn (B.X.); 1918393040@st.gxu.edu.cn (X.P.); 1618306001@alu.gxu.edu.cn (B.C.); 1718306003@alu.gxu.edu.cn (Z.L.); Shenshuibao@gxu.edu.cn (S.S.); 2 School of Education Science, Baise University, Baise 533000, China; nongsiwei2021@foxmail.com

**Keywords:** piglets, microbiota, diarrhea rate, antioxidant capacity, ileum

## Abstract

**Simple Summary:**

The effects of dietary hydrolyzed Chinese gallnut tannic acid (GCT) as a replacement for ZnO were investigated on weaned piglets. A total of 72 piglets (31 ± 1 day) were selected and divided randomly into two groups: a control group, with a basal diet of + 1600 mg/kg ZnO; and a treated group, with a basal diet of + 1899.5 mg/kg GCT. The diarrhea rate of piglets in the treated group declined on days 14–21 than in the control group. Additionally, we found GCT can reduce the crypt depth of the ileum and improve antioxidant capacity. High throughput sequencing showed that the GCT increased the richness of bacteria (*Lachnospiraceae*, *Prevotella,* and *Lactobacillus amylovorus*) associated with the degradation of cellulose and hemicellulose. These data indicate that 1899.5 mg/kg GCT could be an alternative for 1600 mg/kg ZnO in the diet of piglets.

**Abstract:**

The effects of dietary hydrolyzed Chinese gallnut tannic acid(GCT) as a replacement for ZnO were investigated on weaned piglets. A total of 72 weaned piglets at 31 ± 1 day (six replicate pens per treatment with six piglets per pen) were selected and divided randomly into two groups: a control group, with a basal diet of + 1600 mg/kg ZnO; and a treated group, with a basal diet of + 1899.5 mg/kg GCT. Data analysis showed that the significance of average daily gain and average daily feed intake between the two groups was *p* = 0.731 and *p* = 0.799, respectively. Compared with the control group, the diarrhea rate of piglets in the treated group underwent no noticeable change on days 0–7 (*p* = 0.383) and 7–14 (*p* = 0.263), but decreased significantly on days 14–21 (*p* < 0.05). Additionally, we found GCT can reduce the crypt depth of the ileum and improve its antioxidant capacity (*p* < 0.05). High throughput sequencing showed that GCT increased the richness of the bacteria *Lachnospiraceae* (*p* = 0.005), *Prevotella_2* (*p* = 0.046) and *Lactobacillus amylovorus* (*p* = 0.081), which are associated with the degradation of cellulose and hemicellulose. The study indicated that 1899.5 mg/kg GCT could be an alternative for 1600 mg/kg ZnO in the diet of piglets.

## 1. Introduction

Weaning can bring enormous stress to piglets, leading to a decline in piglet growth performance [1]. In recent years, high doses of ZnO have been used to reduce the harm of weaning to piglets [2]. However, the demand for zinc in piglets is 80–100 mg/kg [3], and piglets cannot absorb zinc completely, which leads to a large amount of zinc discharged from the body, causing potential harm to the environment [4]. Announcement No.2625 of the Ministry of Agriculture of China stipulates that the maximum allowable concentration of ZnO in piglets’ diet is 1600 mg/kg. In 2017, the CVMP intends to prohibit the use of high-dose ZnO in piglets within 5 years. Therefore, it is necessary to find a potential alternative to ZnO to maintain the normal growth of weaned piglets.

Some studies have found that tannic acid has good physiological functions: bacteriostasis, anti-diarrhea, anti-oxidation, and anti-inflammation [5,6,7]. However, tannic acid contains a large number of hydroxyl groups [8], which can easily combine with proteins and digestive enzymes to form insoluble complexes [9]. Sathe et al. also found that tannic acid can inhibit pepsin activity [10]. We choose to add stearic acid to hydrolyzed Chinese gallnut tannic acid and to reduce the combination between hydrolyzed Chinese gallnut tannic acid and pepsin. A recent study showed that a dietary supplement of 0.2% tannic acid could effectively alleviate diarrhea in weaned piglets, which may be achieved by improving the integrity and function of intestinal barrier [11]. In this study, we compared the production performance, diarrhea rate, antioxidant capacity, gut morphology, and intestinal flora of two groups of piglets to explore whether 1899.5 mg/kg GCT (0.2%) could replace 1600 mg/kg ZnO as a new piglet feed.

## 2. Materials and Methods

### 2.1. Diets for Piglets

Hydrolytic Chinese gallnut tannic acid was purchased from Ropeedar Biotechnology Co., Ltd. (Shaoxing, Zhejiang, China), which was derived from Chinese gallnut. The hydrolytic tannic acid of *Galla chinensis* was wrapped by stearic acid, and the effective content of tannic acid was 70.38% (calculated by absolute dry weight).

### 2.2. Animals and Management

The Animal Experimental Ethics Committee of Guangxi University approved the experiment (Gxu2018-052). Seventy-two weaned piglets (average initial weight 10 ± 0.2 kg) of 31 days old were randomly divided into two groups: (1) A control group, with a basal diet including + 1600 mg/kg ZnO; ZnO and premix were mixed into diet. (2) A treated group, with a basal diet including + 1899.5 mg/kg GCT (0.2%); tannic acid was evenly added to the premix for feeding.

The whole experiment lasted 21 days. Diets were formulated according to nutritional requirements recommended by NRC (2012) and adjusted according to the situation [3]; detailed information is shown in Table 1. During the experiment, weaned piglets drank water freely and fed regularly 4 times per day. The weight of every piglet was recorded before fasting at 07:00 a.m. in the morning every week, as was the daily feed intake of each pen. Finally, we calculated an average value to obtain the average daily gain (ADG), average daily feed intake (ADFI), FCR (= average daily feed intake (ADFI)/average daily gain (ADG)), and diarrhea rate. Diarrhea monitoring was assessed by fecal score, recorded daily using the score scheme previously proposed by Girard et al. [7].

### 2.3. Sampling and Collection

Blood samples were taken from the jugular vein and put into a heparin anticoagulant tube quickly before feeding (08:00 a.m.) on day 21st. A total of 72 blood samples were collected, 3 of which showed hemolysis due to mis-operation, so only 69 samples were assayed. They were centrifuged at 3000 rpm at 4 °C for 15 min to collect serum and stored in a refrigerator at −80 °C to assay the malondialdehyde (MDA), glutathione (GSH), superoxide dismutase (SOD) and D-lactic acid in serum. MDA, GSH and SOD were assayed with commercial assay kits (Jiangsu Yutong Biological Technology Co., Ltd., Yancheng, China). All blood samples were collected and assayed according to the kit instructions.

### 2.4. Analysis of Intestinal Morphology and Intestinal Flora

We collected 1–2 cm segments of the middle of duodenum, jejunum, and the end of ileum, and put them into a formalin solution for hematoxylin-eosin staining. The pictures were measured using 40× field of view by a light microscope with a computer-assisted morphometric system. We made the tissue fill the entire field when taking pictures, and ensured that the background light of each photo was consistent. Five intact villi and the crypt near the villus were selected for each slice and measured by Image-Pro Plus 6.0 software.

Pigs were euthanized by the intravenous injection of pentobarbital sodium (lethal dose). The cecal contents were collected by gently squeezing the lumen contents from the tissue into the sterile collection tube, then stored in refrigerator at −80 °C to measure the changes of intestinal flora. Genomic DNA isolation of cecal contents was performed according to the instructions of a DNA Stool Mini Kit (Qiagen, Hilden, Germany). DNA isolation was performed by 2% agarose gel electrophoresis. The bacterial universal V3-V4 region of the 16S rRNA gene was amplified according to polymerase chain reaction barcoded primers 515F (5′-ACTCCTACGGGAGGCAGCAG-3′) and the reverse primer 806R (5′-GGACTACHVGGGTWTCTAAT-3′). PCR was run at 95 °C for three minutes to denaturation, followed by twenty seven cycles of 95 °C for thirty seconds, annealing at 55 °C for thirty seconds, 72 °C for forty-five seconds and a final extension at 72 °C for ten minutes. Briefly, paired-end sequencing was performed on the Illumina MiSeq platform at the Majorbio Bio-Pham Technology (Shanghai, China).

### 2.5. Statistical Analysis

Analysis was conducted by Excel of Microsoft (Redmond, WA, USA, Office 2019) and analyzed by SPSS 19.0 (SPSS Inc., Chicago, IL, USA, 2009). Firstly, the data were evaluated through the Shapiro–Wilk test to check whether the distribution of the variables exhibited a normal distribution. Then, the variables that showed a normal distribution and a non-normal distribution were analyzed by the independent sample *t*-test and the Kruskal–Wallis test. Statistical significance was set at *p* < 0.05 and tendencies at 0.05 ≤ *p* ≤ 0.10.

## 3. Results

### 3.1. Production Performance

The results presented in Table 2 show the influence of GCT on the production performance of weaned piglets. Compared with the ZnO diet, the addition of GCT in their diet had no influence on the ADFI (*p* = 0.799), ADG (*p* = 0.731) and FCR of weaned piglets (*p* = 0.411).

### 3.2. Diarrhea Rate

The Table 3 showed the diarrhea rate of piglets. Compared with the ZnO diet, the GCT had no effect on diarrhea rate of weaned piglets on days 0–7 (*p* = 0.383) and 7–14 (*p* = 0.263), but reduce the diarrhea rate of piglets on days 14–21 (*p* = 0.049).

### 3.3. Antioxidant Capacity

The data of Table 4 showed the activity of GSH, MDA, and SOD in serum. The activity of GSH (*p* = 0.012) and SOD (*p* = 0.036) in the serum of the piglets increased in the treated group significantly compared with the CON. The activity of MDA in the serum of the piglets in the treated group decreased (*p* = 0.032) compared with the ZnO diet.

### 3.4. Intestinal Tissue Morphology and Intestinal Barrier

The effect of the GCT supplementation on tissue morphology and the intestinal barrier of the gut are shown in Table 5. Compared with the ZnO diet, the crypt depth of the ileum decreased (*p* = 0.036). In addition, D-lactate acid in the serum of the piglets decreased (*p* = 0.004) further in the treated group than in the CON.

### 3.5. Changes of Intestinal Flora

Analyzing the samples at OTU (operational taxonomic unit) level, it was found that there was no difference (*p* > 0.05) in community richness, evenness, or diversity between the ZnO diet and the hydrolyzed Chinese gallnut tannic acid diet (Table 6).

**Figure 1:**
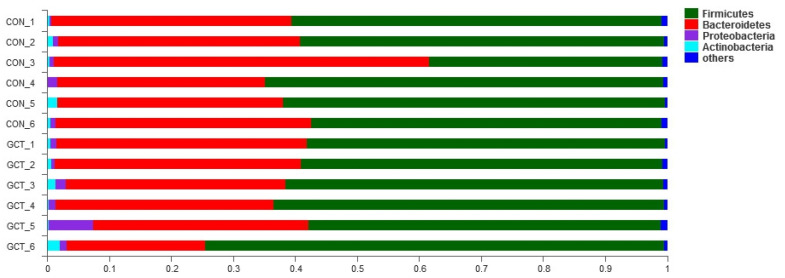
Distribution of piglets’ cecal microflora at the phylum level. The stacked histogram shows the relative abundance of each cecal sample at the phylum level. CON, ZnO diet; GCT, hydrolyzed Chinese gallnut tannic acid diet.

**Figure 2:**
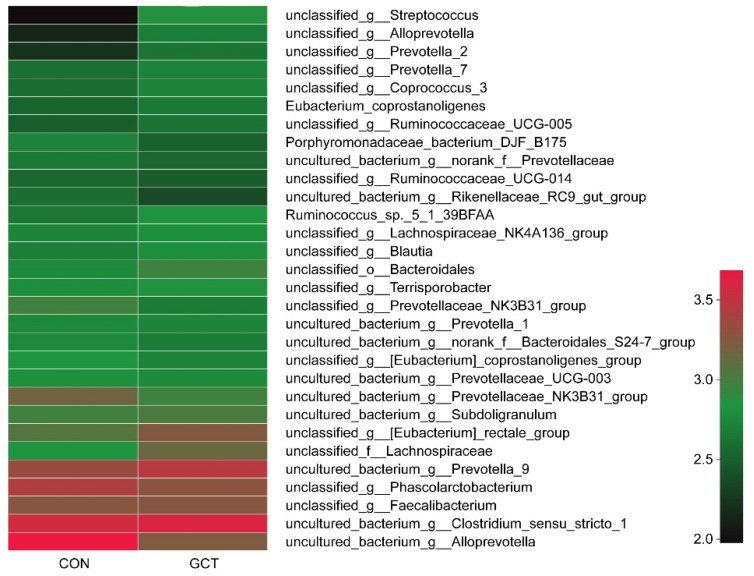
Heatmap shows the top 30 species of bacteria in the cecum of piglets. CON and GCT represent the control group and GCT-treated group, respectively. Numbers represent individual animals.

Distribution of piglets’ cecal microflora is shown in Figure 1 (at the phylum level); Bacteroides and Sclerenchyma represent the main microbial communities in the cecum of piglets. Clustering the cecum samples showed that the dominant communities in the control group were *Alloprevotella* and *Clostridium sensu stricto 1*. The dominant communities in the GCT-treated group were *Clostridium sensu stricto 1* and *Prevotella 9* (Figure 2).

**Figure 3:**
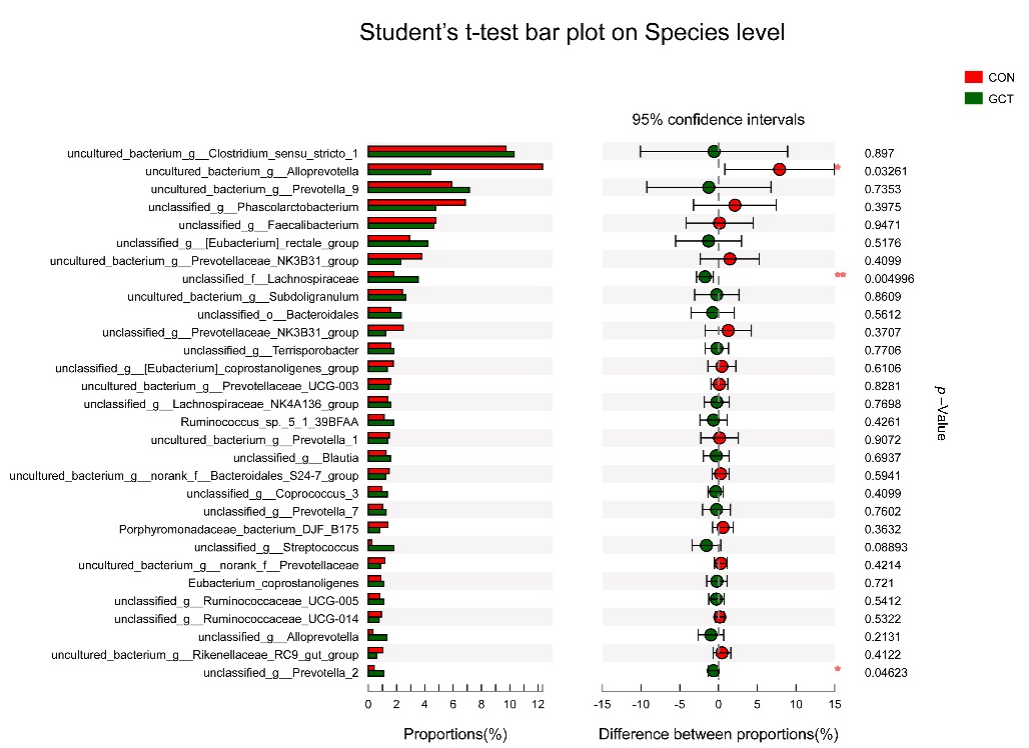
The microbial richness of cecum community of piglets ranked according to the top 30 at species level. Red bar represents the control group, green bar represents the GCT-treated group. The rightmost side is *p* -Value, * means 0.01 < *p* < 0.05, ** means 0.001 < *p* < 0.01.

**Figure 4:**
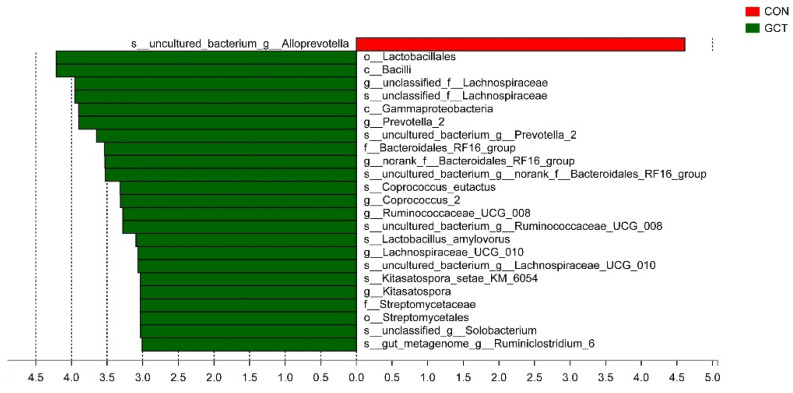
Differences between bacterial taxa in control group and GCT-treated group. The histogram shows differentially rich bacteria in the control group and GCT-treated group ranked by linear discriminant analysis (LDA) scores. Red bars represent the bacteria in the control group are richer than in the GCT-treated group. Green bars represent the bacteria in the GCT-treated group are richer than in the control group; c: class level, o: order level, f: family level, g: genus level, s: species level.

The difference in richness of the top 30 bacteria of the cecum community in piglets was analyzed at species level, and it was found that GCT reduced (*p* < 0.05) the richness of *Alloprevotella* and improved (*p* < 0.05) the richness of *Prevotella_2* and *Lachnospiraceae* in the cecum of the piglets (Figure 3). All the differences of microbes in the cecum of piglets between the control group and treated group were analyzed at phylum to species level; there were 11 species of microbes with differences (*p* < 0.05) between the CON and GCT group at species level (Figure 4).

## 4. Discussion

It has always been a controversial issue whether tannic acid can improve the diet utilization rate of piglets. Tannic acid has long been considered an “anti-nutritional” factor. However, Bee et al. have found that hydrolyzed tannins seem to have less impact on growth performance than concentrated tannins [12]. Kotrotsios et al. have found that high doses of condensed tannin (9.7 g/kg) can reduce ADFI for piglets [13]. Although high-dose hydrolyzed tannin (4.5 g/kg) fed piglets can also reduce the FCR, it has no effect on ADFI and ADG [14]. We used stearic acid to wrap hydrolyzed Chinese gallnut tannic acid to reduce its effect on pepsin, but the effect of tannic acid on digestive enzymes in the small intestine is unavoidable [15]. The results show no influence on ADG, ADFI and FCR between the two diets. It is suggested that some beneficial changes may have taken place in the intestine, but these changes are still unknown.

A previous review explained that tannic acid hydrolysis can reduce the rate and duration of diarrhea in piglets [16]. Our study also proved this point: compared with the control group, we found that GCT can reduce the diarrhea rate, and the effect is increasingly obvious with the extension of time. Within the first 14 days, the duration of diarrhea in the treatment group was generally 1–2 days, while that in the control group was about 3 days, which is consistent with the previous report [16]. From 14 to 21 days, the piglets in the control group still had diarrhea, which is different from the results reported by Madec [17]. We must, however, take into account that Madec’s research was conducted in a pathogen-free environment, not in traditional pig farms.

Piglets are prone to stress in the early stage of weaning; the immune organs in the body are not fully developed and mature, and coupled with the stress of the feeding environment, it is easy to produce free radicals, which reduce the body’s resistance and growth performance [18]. MDA is a marker of oxidative stress, which reflects the degree of free radical attack on body cells. SOD and GSH are important components of the antioxidant defense system, which can scavenge free radicals or lipid peroxides. Therefore, we also studied the effect of GCT on the antioxidant capacity of piglets. Ye et al. found that tannic acid can improve the antioxidant capacity of piglets [19]. The data showed that the GCT reduced the MDA and increased the SOD and GSH, improving the antioxidant capacity of piglets and improving weaning stress.

One limitation of our study is that we did not assess the content of butyric acid in the piglets’ intestines. In vitro experiments showed that the decomposition products of tannic acid contain butyric acid [13]. Butyric acid is the preferred energy substrate for ileal mucosa [20]. It has been proved that 2.25 mg/kg tannic acid can reduce the depth of ileal crypt and increase the content of butyric acid in intestine [13]. In agreement with Kotrotsios et al., in our experiment, the depth of ileal crypt decreased in the treated group. The crypts of the small intestine have a secretory function, so reducing its surface may help to reduce the severity of diarrhea in weaned piglets [21]. Ampting et al. found that tannic acid was beneficial for the intestinal barrier of piglets [22]. Lactic acid is one of the main products of rapid fermentation of an animal’s stomach, and a large amount of lactic acid can cause subacute rumen acidosis in ruminants [23]. The production of lactic acid is negatively correlated with animal health; it can pass through the gastrointestinal wall, dissolve in the blood, and finally lead to an increase in the blood [24]. In agreement with these reports, we found that GCT could significantly reduce D-lactic acid in serum. It is suggested that GCT can improve the intestinal barrier.

When we analyzed the samples at OTU level, we found no significant influence on the community richness, evenness, or diversity of the two groups. Most of the bacteria in Bacteroidetes and Firmicutes are anaerobic, and the cecum is an absolutely anaerobic environment, so it is predictable that Bacteroidetes and Firmicutes account for about 95% of the cecum bacteria, regardless of the treatment group. This result is similar to that reported by Wang et al. [25]. When we analyzed the samples at the species level, we detected that the richness of some bacteria in the GCT diet decreased, such as the opportunistic pathogen *Allprevotella*. Therefore, GCT could reduce opportunistic pathogens in the cecum of piglets. When piglets are newly weaned, their food is changed from sow milk to feed containing cellulose and hemicellulose (soybean and corn). *Prevotella* is the leader in the degradation of hemicellulose in piglets’ cecum [25,26]. *Lachnospiraceae* also has a strong ability to degrade polysaccharides, even cellulose and hemicellulose [27]. In our experiment, GCT significantly increased the richness of *Lachnospiraceae* and *Prevotella* in piglets’ cecum. In addition, some articles have reported that *Lachnospiraceae* can reduce the incidence of colitis by reducing the colonization of Clostridium difficile in the intestine [28,29]. *Lactobacillus amylovorus* has a strong ability to hydrolyze starch, and its hydrolysis efficiency is 10 times higher than that of *Lactobacillus plantarum* [30]. In this experiment, GCT increased the richness of *Lactobacillus amylovorus* in the cecum of piglets. Overall, GCT may support weaned piglet production performance by promoting the growth of bacteria specialized in the degradation of polysaccharides in the cecum. These results indicate that 1899.5 mg/kg GCT could be an alternative to 1600 mg/kg ZnO in the post-weaning diets of piglets.

## 5. Conclusions

We found that GCT could reduce the depth of ileal crypt and oxidative stress in piglets. Meanwhile, GCT improve intestinal barrier and reduce diarrhea rate. Furthermore, GCT could improve the richness of bacteria associated with the degradation of cellulose and hemicellulose. Thus, adding GCT could be an alternative to ZnO in the post-weaning diets of piglets.

## Figures and Tables

**Table 1:** Ingredients and nutrient levels for weaned piglets.

Composition (%)	Content	Nutrient Levels of Basal Diets	Content
Corn	56.5	Crude protein	18.2
Soybean meal	10	Crude fat	6.2
Puffed soybean	8	Crude fiber	2.5
Fermented soybean meal	5	Crude ash	5
Flour	5	Lysine	1.35
Fish meal	4	Chloride	0.6
Soybean oil	3.5	P	0.55
Glucose	2.5	Ca	0.8
Whey powder	2.5	Digestive energy (MJ/kg)	14.23
Premix ^A^	3		

^A^ Provided per kg diet: 150 mg of Fe (as FeSO_4_), 85 mg of Cu (as CuSO_4_), 80 mg of Mn, 10,000 IU of vitamin A, 1200 IU of vitamin D_3_, 30 IU of vitamin E, 1.5 mg of vitamin B_1_, 3.5 mg of vitamin B_2_, 1.4 mg of vitamin B_6_, 0.03 mg of vitamin B_12_, 30 mg of nicotinic acid, 15 mg of D-pantothenic acid, 0.14 mg of biotin.

**Table 2:** Effect of GCT on production performance of weaned piglets.

Item	CON	GCT	SEM	*p* Value
Initial weight (kg)	10.10	10.05	0.047	0.615
Final weight (kg)	18.83	18.95	0.075	0.469
ADG (g/day)	418.86	420.22	2.50	0.731
ADFI (g/day)	748.90	752.96	5.52	0.799
FCR	1.81	1.79	0.008	0.411

CON: ZnO diet; GCT: Hydrolyzed Chinese gallnut tannic acid diet; ADG: Average daily gain; ADFI: Average daily feed intake; FCR = ADG/ADFI.

**Table 3:** Effect of GCT on diarrhea rate during the post weaning period in piglets.

Item	CON	GCT	SEM	*p* Value
0–7 days	3.17%	3.97%	0.0095	0.383
7–14 days	2.78%	1.98%	0.0059	0.263
14–21 days	1.19%	0	0.0031	0.049

CON: ZnO diet; GCT: Hydrolyzed Chinese gallnut tannic acid diet.

**Table 4:** Effect of GCT on antioxidant capacity of weaned piglets.

Item	CON	GCT	SEM	*p* Value
GSH (mg/mL)	5.57	6.43	0.19	0.012
MDA (nmol/mL)	4.15	3.58	0.14	0.032
SOD (U/mL)	97.16	105.47	2.06	0.036

CON: ZnO diet; GCT: Hydrolyzed Chinese gallnut tannic acid diet; GSH: Glutathione; MDA: Malondialdehyde; SOD: Superoxide dismutase.

**Table 5:** Intestinal barrier and intestinal morphology of weaned piglets.

Item	CON	GCT	SEM	*p* Value
Villus height (um)				
Duodenum	454.25	401.02	15.63	0.088
Jejunum	422.44	410.70	16.99	0.747
Ileum	384.67	399.66	14.95	0.639
Crypt depth (um)				
Duodenum	142.70	148.20	2.90	0.367
Jejunum	159.04	150.06	4.02	0.284
Ileum	144.69	128.25	4.08	0.036
D-lac	1288	1106	36.29	0.004

CON: ZnO diet; GCT: Hydrolyzed Chinese gallnut tannic acid diet; D-lac: D-lactic acid; SEM: Standard error of the mean.

**Table 6:** Effects of GCT on community richness, evenness, and diversity of cecum intestinal flora.

Item	CON	GCT	SEM	*p* Value
Community richness				
Sobs	394.83	436.33	13.04	0.114
Ace	435.11	474.88	13.13	0.136
Chao	448.93	482.20	13.54	0.236
Community evenness				
Heip	0.15	0.165	0.0087	0.294
Simpsoneven	0.058	0.07	0.0057	0.358
Shannoneven	0.68	0.7	0.0098	0.251
Community diversity				
Shannon	4.05	4.26	0.072	0.152
Simpson	0.047	0.036	0.0046	0.236

CON: ZnO diet; GCT: Hydrolyzed Chinese gallnut tannic acid diet; Sobs, Chao and Ace are indices reflecting cecum community richness; Simpsoneven, Shannoneven and Heip are indices of community evenness; Shannon and Simpson are indices of community diversity.

## Data Availability

The data presented in this study are available on request from the corresponding author. The availability of the data is restricted to investigators based in academic institutions.
